# Development and validation of new measure to assess social connection in long‐term care residents: the SONNET study

**DOI:** 10.1002/alz.084584

**Published:** 2025-01-09

**Authors:** Andrew Sommerlad

**Affiliations:** ^1^ Camden and Islington NHS Foundation Trust, London, United Kingdom; University College London, London United Kingdom

## Abstract

**Background:**

Long‐term care (LTC) home residents may be isolated or lonely. Social connection is important for their physical, mental and cognitive health, quality of life and care. However, measuring social connection in LTC residents is challenging and there are no existing measures with adequately established psychometric properties. We aimed to develop and test a new measure of social connection for LTC residents.

**Method:**

The SONNET study consisted of three projects with data collection carried out in parallel in LTC homes in Canada and the UK. First, we conducted a systematic review of existing measures of social connection in LTC residents, finding 62 studies reporting on 38 measures; none had sufficient evidence to be recommended for use. Second, we conducted qualitative interviews about key components of social connection with 67 LTC residents with and without dementia, family caregivers, staff, and clinicians. Third, we used these findings to inform candidate items for a scale with resident‐rated and proxy‐rated versions based on measurable domains and indicators of social engagement and social connectedness. We refined and reduced these through patient and public engagement and pilot interviews. We pre‐tested the long‐form scale in LTC residents for usability, missing data, response option distribution, internal consistency and factor structure. We then field‐tested the final SONNET measure for reliability, content and construct validity.

**Result:**

We tested the SONNET measure with 111 LTC residents, including 40 with dementia (mean age 85.3y.) The measure was feasible with complete data in 86.8% of resident‐rated responses and 98.8% of carer‐rated responses. Internal consistency was high (resident‐rated scale α = 0.85, proxy‐rated α = 0.83) Exploratory factor analysis showed that SONNET scale sum scores are reliable in measuring overall social connection, and subdomains ‘social engagement’ and ‘social connectedness’.

**Conclusion:**

The SONNET measure assesses key domains of social connection in LTC residents and there is preliminary evidence of its reliability and validity. Further studies of its psychometric properties in independent samples of LTC residents including longitudinal data to assess its sensitivity to detect change and predictive and cross‐cultural validity are required. The SONNET measure will be freely available for researchers to use.

